# Preoperative Radiotherapy and Wide Resection for Soft Tissue Sarcomas: Achieving a Low Rate of Major Wound Complications with the Use of Flaps. Results of a Single Surgical Team

**DOI:** 10.3389/fsurg.2017.00079

**Published:** 2018-01-22

**Authors:** Lester Wai Mon Chan, Jungo Imanishi, Damien Glen Grinsell, Peter Choong

**Affiliations:** ^1^Department of Orthopaedics, St. Vincent’s Hospital, Melbourne, VIC, Australia; ^2^Department of Orthopaedic Surgery, Tan Tock Seng Hospital, Singapore, Singapore; ^3^Department of Plastic Surgery, St Vincent’s Hospital, Melbourne, VIC, Australia; ^4^Department of Surgery, University of Melbourne, Melbourne, VIC, Australia

**Keywords:** soft tissue sarcoma, preoperative radiotherapy, wound complication, reconstructive surgery, flap reconstruction

## Abstract

**Background:**

Surgery in combination with radiotherapy (RT) has become the standard of care for most soft tissue sarcomas. The choice between pre- and postoperative RT is controversial. Preoperative RT is associated with a 32–35% rate of major wound complications (MWC) and 16–25% rate of reoperation. The role of vascularized soft tissue “flaps” in reducing complications is unclear. We report the outcomes of patients treated with preoperative RT, resection, and flap reconstruction.

**Patients and methods:**

122 treatment episodes involving 117 patients were retrospectively reviewed. All patients were treated with 50.4 Gy of external beam radiation. Surgery was performed at 4–8 weeks after completion of RT by the same combination of orthopedic oncology and plastic reconstructive surgeon. Defects were reconstructed with 64 free and 59 pedicled/local flaps.

**Results:**

30 (25%) patients experienced a MWC and 17 (14%) required further surgery. 20% of complications were exclusively related to the donor site. There was complete or partial loss of three flaps. There was no difference in the rate of MWC or reoperation for complications with respect to age, sex, tumor site, previous unplanned excision, tumor grade, depth, and type of flap. Tumor size ≥8 cm was associated with a higher rate of reoperation (11/44 vs 6/78; *P* = 0.008) but the rate of MWC was not significant (16/44 vs 14/78; *P* = 0.066).

**Conclusion:**

The use of soft tissue flaps is associated with a low rate of MWC and reoperation. Our results suggest that a high rate of flap usage may be required to observe a reduction in complication rates.

## Introduction

Surgery in combination with radiotherapy (RT) has become the standard of care for soft tissue sarcomas (STSs) of the extremity and trunk (1–3), however, the choice between preoperative vs postoperative RT continues to be controversial (4, 5). Preoperative RT is delivered to well-oxygenated, undisrupted tissues, allowing lower doses to be administered to a smaller field and resulting in reduced rates of long-term radiation toxicity with similar rates of local control (5, 6). The main disadvantage of preoperative RT is the high rate of postoperative wound complications. The reported rates are variable and partially dependent on the definition used; the rate of major wound complications (MWCs) as defined by the Canadian Clinical Trials Group is around 32–35% with 16–25% of patients requiring further surgery for complications (4, 7–13).

On principle, the requirements for predictable wound healing are low wound tension, minimization of dead space, and well-vascularized tissue. All three factors are negatively affected in the preoperatively irradiated sarcoma wound. Theoretically, all these factors can be improved with the use of vascularized soft tissue flaps (14). While flaps are commonly used for the closure of soft tissue defects, only one historical series has demonstrated a reduction in the rate of wound complications (15) with the majority of the literature reporting no difference in outcomes (10, 11, 13, 16–19).

Preoperative RT followed by wide surgical resection is the preferred management of STSs at our center (16). In 2002, we published the results of our STS protocol. At that time, free and pedicled flaps were used in only 35% of cases. 44% of patients developed postoperative wound complications and 23% required further surgery. Since that time, we have increased our utilization of soft tissue flaps in an attempt to achieve more predictable wound healing. We currently employ flaps in 79% of cases (source: 2011–2015 data. Decision Support Unit, St. Vincent’s Hospital, Melbourne).

The aim of this study is to report the wound complication outcomes of a strategy of preoperative RT, resection, and flap reconstruction.

## Patients and Methods

This was a retrospective study of consecutive cases performed by the same combination of orthopedic oncology surgeon (PC) and plastic surgery reconstructive surgeon (DG). Institutional review board approval was obtained prior to the study (HREC number: QA106/15). Subjects were identified from the institution database. Inclusion criteria were patients with STSs and other locally aggressive soft tissue tumors treated with a standard protocol of preoperative RT, surgical resection and soft tissue reconstruction with a flap. Only patients operated on by the two senior authors were included. The cohort included patients from the private and public hospital systems. Inpatient and outpatient hospital medical records and electronic medical records from private consulting rooms were reviewed for complications. Patients who were closed without a flap and those with missing data were excluded.

Patients underwent surgery between April 2008 and June 2015. During the study period, there were 512 episodes of definitive surgery for STS identified from our institution database. Of these, 129 episodes were performed by the two senior authors. Three episodes were excluded as closure was achieved without a flap and four episodes were excluded because of missing data. In total, there were 122 treatment episodes involving 117 patients that were eligible for review. Five patients were included twice with soft tissue metastases distant to the original resection site that were treated with a separate course of preoperative RT, wide resection, and flap reconstruction.

The patient and disease characteristics are shown in Table 1.

**Table 1:** Patient characteristics.

Parameter	No. (%), *n* = 122
**Mean age**	58 years (range: 15–89)
**Sex**	
Male	64 (52)
Female	58 (48)
**Diagnosis**	
Undifferentiated pleomorphic sarcoma	39 (32)
Liposarcoma	26 (21)
Myxofibrosarcoma	18 (15)
Synovial sarcoma	9 (7)
Leiomyosarcoma	6 (5)
Solitary fibrous tumor	5 (4)
Fibromyxoid sarcoma	4 (3)
MPNST	4 (3)
Clear cell sarcoma	3 (2)
Others	8 (7)
**FNCLCC grade**	
I/borderline malignancy	19 (16)
II	44 (36)
III	59 (48)
**Tumor size**	
<8 cm	78 (64)
≥8 cm	44 (36)
**Tumor depth**	
Superficial	32 (26)
Deep	90 (74)
**Tumor site**	
Upper limb	19 (16)
Lower limb	85 (70)
Axial	18 (15)
**Previous surgery to tumor bed**	
None	62 (51)
Intralesional/marginal	54 (44)
Wide margin	7 (6)

*MPNST, Malignant Peripheral Nerve Sheath Tumor; FNCLCC, Fédération Nationale des Centres de Lutte Contre le Cancer*.

After confirmation of diagnosis at our sarcoma multidisciplinary meeting, patients were treated with 50.4 Gy of external beam radiation delivered in 28 fractions over five and a half weeks. Surgery was performed at 4–8 weeks post completion of RT to allow subsidence of inflammation. We have previously reported our protocol and experience with preoperative RT (16, 20).

The definition of MWC was that of the NCI Canadian Clinical Trials Group (21). This included any of the following criteria occurring within 120 days of surgery: a secondary operation under general or regional anesthesia for wound repair, an invasive procedure without general or regional anesthesia (mainly aspiration of seroma), readmission for wound care such as intravenous antibiotics, and wound packing deep to dermis in an area >2 cm with or without prolonged dressings >6 weeks from wound breakdown.

### Statistical Analysis

Statistical analysis was performed using JMP^®^ version 10 (SAS Institute Inc., Cary, NC, USA). The association of factors with MWC and reoperation for complications were analyzed using chi-squared or Fisher’s exact test. *P* < 0.05 was regarded as statistically significant.

## Results

There were 64 free flap and 59 pedicled or local flaps utilized in 122 treatment episodes. One patient had reconstruction with a combination of two flaps. The types of free and pedicled or local flaps employed are shown in Tables 2 and 3 respectively.

**Table 2:** Type of free flap used.

Free flap type	No.
Anterolateral thigh	18
Latissimus dorsi	11
Gracilis	12
Parascapula/scapula	10
Rectus	5
Groin	3
Tensor fasciae latae	2
Anteromedial thigh	1
Radial artery forearm	1
Fibula osteocutaneous	1

**Total**	**64**

**Table 3:** Type of pedicled/local flap.

Pedicled/local type	No.
Anterolateral thigh	14
Latissimus dorsi	10
Rectus	8
Gluteal perforator	4
Gastrocnemius/soleus	3
Parascapular/scapula	3
Pectoralis major	2
Sartorius	2
Thoracodorsal artery perforator	2
Medial thigh perforator	2
Radial artery forearm	1
Medial arm	1
Superficial inferior epigastric artery	1
Internal mammary artery perforator	1
Lumbar artery perforator	1
Local deepithelialized skin	2
Random pattern skin flap	2

**Total**	**59**

**Figure 1:**
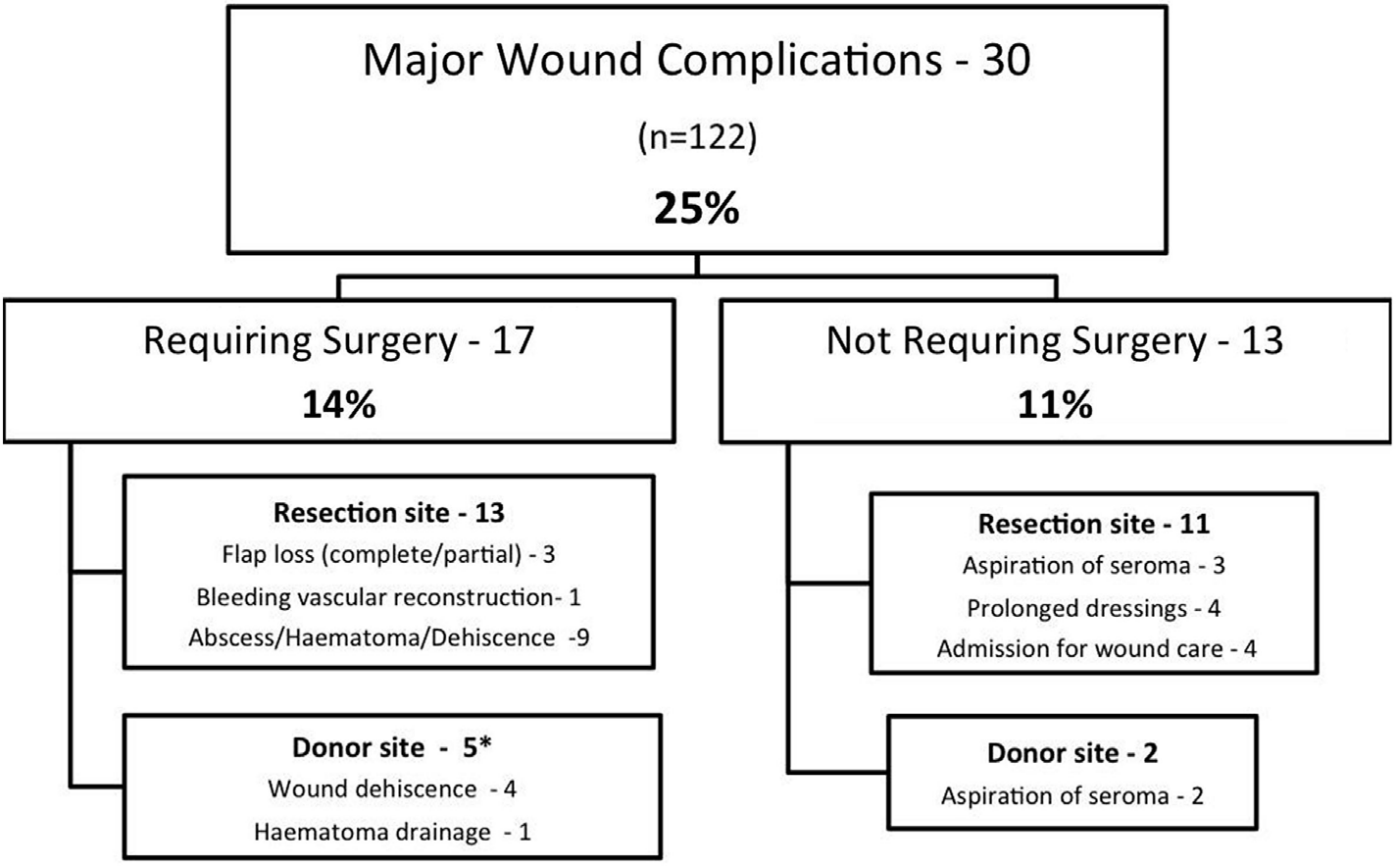
Summary of major wound complications*. One patient had resection and donor site complications.

A summary of MWCs is shown in Figure 1. A total of 30 patients (25%) experienced a MWC; 17 patients (14%) required surgery for correction of complications. MWCs affected the irradiated resection site in 24 patients and the donor site in seven patients (one patient had complications affecting the donor and resection site).

For patients requiring further surgery, the average number of additional surgical procedures was 1.5 (range 1–3). An analysis of the association of MWC with patient and disease variables is shown in Table 4. There was no difference in the rate of MWC or reoperation for age, sex, site of tumor, previous unplanned excision, FNCLCC grade, tumor depth, and type of flap. Tumor size ≥8 cm was associated with an increased rate of reoperation (*P* = 0.008) but no significant difference in MWC (*P* = 0.066).

**Table 4:** Association of MWC and reoperations with patient and disease variables.

Variable	Major wound complication (MWC), no. (%)	*P*-value	Reoperation, no. (%)	*P*-value
**Age**				
≥60	12/56 (21.4)	0.46	9/66 (13.6)	0.91
<60	18/66 (27.3)		8/56 (14.3)	
**Sex**				
M	16/64 (25)	0.91	9/64 (14.1)	0.97
F	14/58 (24.1)		8/58 (13.8)	
**Site**				
Upper limb	6/19 (31.6)	0.24	3/19 (15.8)	0.47
Lower limb	22/85 (25.9)		13/85 (15.3)	
Axial	2/18 (11.1)		1/18 (5.6)	
**Size**				
≥8 cm	16/44 (36.4)	0.066	**11/44 (25)**	**0.008**
<8 cm	14/78 (17.9)		**6/78 (7.7)**	
**Previous unplanned excision**				
Y	11/54 (20.4)	0.33	7/54 (13.0)	0.78
N	19/68 (27.9)		10/68 (14.7)	
**FNCLCC grade**				
0 and 1	3/19 (15.8)	0.40	1/19 (5.3)	0.47
2 and 3	27/103 (26.2)		16/103 (15.5)	
**Deep/superficial**				
Deep	25/90 (27.7)	0.17	13/90 (14.4)	0.78
Superficial	5/32 (15.6)		4/32 (12.5)	
**Type of flap**				
Free flap	19/63 (30.2)	0.33	11/63 (17.5)	0.26
Pedicled/local	11/58 (19.0)		6/58 (10.3)	

*FNCLCC, Fédération Nationale des Centres de Lutte Contre le Cancer*.

*The bold text indicates a statistically significant result P < 0.05*.

There were four patients who required complex revision surgery. Two patients had methicillin-resistant *Staphylococcus aureus* infection with complete or partial necrosis of free flaps. One required debridement of 40% of a free anterolateral thigh flap and the remaining defect was adequately covered with a pedicled medial arm flap and a split skin graft; the second required complete removal of a free gracilis flap that was revised to a pedicled latissimus dorsi flap. Another patient had bleeding from the anastomosis for an innervated latissimus dorsi flap. This was revised once but a further episode of severe bleeding necessitated emergency flap removal. The fourth patient was readmitted for a delayed presentation of a leaking superficial femoral artery vein graft on postoperative day 61. This was resolved with revision of the graft. All severe complications occurred in elderly patients (mean age 82, range 74–89) who had undergone free flap reconstruction.

The remaining 13 patients who underwent secondary surgery required relatively simple procedures; surgery was at the resection site in nine cases and the donor site in four. Resection site procedures included closure of wound dehiscences, skin edge debridements, and open drainage of collections. Donor site complications were typically mild. Three patients had simple wound dehiscences that were debrided and resutured and one patient had a hematoma that was evacuated and closed over a surgical drain.

There were 13 complications not requiring surgery (see Figure 1). Of these, there were five patients requiring percutaneous needle drainage of seromas, four patients with prolonged dressings, and four patients who were admitted to hospital for intravenous antibiotics. All patients with prolonged dressings went on to complete wound healing.

We performed a subgroup analysis of early vs late cases (see Table 5). There were no significant differences in age, sex, size, previous unplanned excision, FNCLCC grade MWC, or reoperation. A higher proportion of free flaps were performed in the early part of the study (free/pedicled-local; 37/23 vs 26/35, *P* = 0.036).

**Table 5:** Subgroup analysis of early vs late cases.

Variable	Early (61)	Late (61)	*P*-value
**Age**			
≥60	33	33	1
<60	28	28	
**Sex**			
M	32	32	1
F	29	29	
**Size**			
≥8 cm	26	18	0.13
<8 cm	35	43	
**Previous unplanned excision**			
Y	29	25	0.33
N	32	36	
**FNCLCC grade**			
0 and 1	8	11	0.62
2 and 3	53	50	
**Type of flap**			
Free flap	37	26	0.036
Pedicled/local	23	35	
**MWC**			
Yes	11	19	0.09
No	50	42	
**Reoperation**			
Yes	6	11	0.30
No	55	50	

*FNCLCC, Fédération Nationale des Centres de Lutte Contre le Cancer*.

## Discussion

Preoperative RT for STSs is associated with a high rate of postoperative wound complications. In 1992, Barwick et al. reported a series of 82 patients, comparing their use of vascularized tissue transfer with direct closure in irradiated wounds. Direct closure was used early in their series and the authors noted a high rate of wound complications. This caused them to adopt a practice of routine plastic surgical consultation before surgery, which led to an increased utilization of flaps. They showed a lower complication rate (19 vs 51%) and fewer secondary procedures (10 vs 35%) with flap reconstruction. The overall flap rate in their study was 52% (15). Since this report more than 20 years ago multiple authors have failed to show a lower complication rate with the use of flaps (4, 10, 11, 13, 16, 17, 19). There are no randomized trials evaluating the use of flaps for sarcoma wounds and retrospective studies suffer from inherent bias against advanced reconstruction techniques; wounds at high risk of failure tend to be treated with flaps whereas small low risk wounds are treated by direct closure, therefore masking any positive effect of flaps.

The present study is a single armed series of recent (2008–2015) STS cases treated with vascularized soft tissue flap reconstruction. It demonstrates a low rate of MWC (25%) and secondary operations (14%) compared to other reports (see Table 6) and suggest that the use of “flaps” may be useful in reducing the rate of wound complications. This series represents modern microsurgical practice and does not include any previously published data on this topic. It also represents a change in reconstructive philosophy compared to that of a previous study conducted at our center in 2002. At that time, flaps were used in 35% of patients; 44% developed a wound complication and 23% required an additional surgical procedure. In an effort to reduce our complication rate, we began routine preoperative consultation with our plastic surgeons. This led to an increase in our utilization of flaps such that we now use them in 79% of cases (16).

**Table 6:** Summary of recent papers investigating complications of preoperative radiation for soft tissue sarcomas (STSs).

Reference	Description	Outcome measure	Complication rate	Reoperation rate	Use of vascularized flaps
O’Sullivan et al. (4)	Randomized control trial of pre- vs postoperative RT for STS	Canadian Trials group	35% preoperative, 17% postoperative^a^	Not reported	28%
Virkus et al. (7)	Retrospective study of preoperative RT	Moderate or major wound complications (MWCs). Seroma aspiration, IV antibiotics not included as complications	22% preoperative^b^	Not reported	13% Flap or split-thickness skin graft
Kunisada et al. (16)	Retrospective study of preoperative RT	Complication—not stated. MWC—requiring a second procedure	44% preoperative	23%	35%
Tseng et al. (19)	Preoperative RT. Retrospective analysis of impact of plastic surgical closure	Canadian Trials group	32% (plastics closure 32%)^a^	Not reported	40%
Cannon et al. (9)	Retrospective analysis of factors influencing wound complications	Similar to Canadian trials group but included “mild” complications not requiring specific intervention	34% preoperative, 16% postoperative	Not reported	22%
Townley et al. (17)	Retrospective analysis of free flap reconstruction in patients with preoperative RT vs postoperative/non-RT control group	Wound complication—not specified.Microsurgical flap complications	50% preoperative, 14% control, 12.5% flap complications	Not reported	100%—only free flap cases reviewed
Baldini et al. (10)	Preoperative RT. Retrospective analysis of predictors of wound complications	Canadian Trials group	35% preoperative^a^	**25%**	27%
Schwartz et al. (12)	Retrospective analysis of wound complications following resection of sarcomas. Included patients with preoperative RT + brachytherapy/IntraopRT	Requiring secondary flap	15% preoperative, 11% postoperative	Not reported	0%—primary flap closure excluded
Rosenberg et al. (13)	Preoperative RT. Retrospective analysis of impact of plastics closure	Canadian Trials group	32% (plastics closure 28%)^a^	**16% (11%)**	22%
Moore et al. (11)	Retrospective analysis of risk factors for WC after sarcoma resection	Surgical intervention or outpatient debridement and negative pressure wound therapy. Seroma aspiration, IV antibiotics not included.	23.8% preoperative^b^, 11.9 postoperative		Unknown
Chan (2017) (Current series)	Preoperative RT with flap reconstruction. Retrospective analysis of complications	Canadian Trials group	25% preoperative RT with flap closure^a^	**15%**	79%

*^a^Canadian Trial Group criteria highlighted in bold*.

*^b^The definition of wound complication for Virkus and Moore did not include aspiration of seroma or admission for IV antibiotics. The comparative wound complication rate in our study was 17%*.

We note that the rate of flap use is low in most series evaluating pre-operative RT for sarcomas (13–40%). We consider this to reflect a difference in philosophy where flaps are used to achieve wound closure rather than to reduce wound complications. By contrast, the indication for flaps at our center is not purely to achieve wound closure; but rather to reduce tension, dead space, and introduce vascularized tissue into wounds that we previously may have closed primarily (14, 16). This was also the intent of flaps reported by Barwick et al. (15). We, therefore, emphasize the difference between wounds that *can* be closed primarily vs wounds that *should* be closed. By reducing our threshold for the use of flaps both Barwick et al. and the current study have been able to achieve lower rate of wound complications.

For the purpose of comparison, we used the Canadian Trials Group definition of MWCs as our primary endpoint; however, we recognize that the definition is broad and includes severe wound complications requiring extensive additional surgery alongside milder complications such as seromas requiring needle aspiration or admissions for intravenous antibiotics alone. In order to better define severity, we also reported the number of patients requiring further surgery as a second endpoint. While this is an imperfect surrogate measure of severity (the same wound breakdown might be treated with surgery or dressings according to the practice of the treating surgeon), it is objective and simple to record and compare.

We found that the type of complications in our series reflected the nature of flap surgery with one-fifth of complications being exclusively related to the donor site. We found complications at the non-irradiated donor site generally straightforward to rectify. In this way, flap surgery may trade-off more challenging complications at the resection site for “simple” wound issues at the donor site. The original definition of MWC does not specifically mention donor site complications; for the purpose of this study they were included as MWCs. If donor site complications were excluded, the MWC rate for the irradiated wound would be 20%.

There were three complications related to the microvascular anastomosis in elderly patients with free flaps, this included one case of life threatening bleeding. While neither age nor type of flap was significantly associated with MWC or reoperation, this finding raises some caution for this subgroup.

There were two patients who suffered complete flap necrosis and were subsequently closed with a pedicled flap and direct closure, respectively; both these patients could have been closed initially without a free flap, however, as mentioned previously, the indications for flaps in our series and the choice of flap was made on the basis of reasons other than closure alone (filling of dead space, introduction of vascularized tissue, reduction of wound tension) and influenced by factors, including the size and suitability of donor tissue and potential donor site morbidity. In the first case, a free gracilis was felt to be a more suitable donor for an anterior arm defect with lower donor morbidity than the pedicled latissimus dorsi that was subsequently required for salvage; in the second case, a free latissimus dorsi was used to fill the dead space in a large posterior thigh defect that eventually had to be closed directly. Unfortunately neither of these patients benefited from the theoretical advantages of their flaps.

Previous authors have shown an association of complications with larger, high-grade tumors and lower extremity location (7, 9–13, 19). We did not find any significant differences in MWC or reoperation rates for any of the factors reviewed with the exception of size ≥8 cm. The reasons for this are uncertain; however, a lower rate of complications of itself may make statistical significance more difficult to achieve.

Late complications and functional outcomes were not assessed as part of this study. While this is consistent with the majority of the contemporary literature, a recent study by Miller et al. examined the pattern and timing of MWC in 102 patients with STS treated with multimodal therapy and noted that 45% of MWC occurred after 120 days post resection; of note, 8 out of 10 late complications occurred in patients who were treated with post-operative RT and the authors did not comment on the type and severity of late complications (22). In the present study, all MWCs occurred within 120 days from surgery and we did not detect any new MWCs after this period. We postulate that late complications may be of greater significance in studies involving post-operative RT where wound complications may be induced to occur later, during the post-RT period, rather than in the earlier post-operative period.

Microsurgical flap reconstruction is technically demanding, particularly in the context of pre-operative RT. Previous authors have demonstrated that good outcomes can be achieved in irradiated wounds and that flap outcomes are not adversely affected in experienced hands (17, 23, 24). We attempted to review the effect of experience on MWC and reoperation, however, a subgroup analysis of early vs late cases with respect to MWC, reoperations showed no significant difference in the rate of MWC or reoperation. There was, however, an increase in the use of pedicled/local flaps in the latter half of the study. We postulate that this difference could be due to a clinically but not statistically significant difference in tumor size. Smaller tumors in the latter part of the study (*P* = 0.13) may have resulted in wounds more amenable to pedicled flap closure. This difference in tumor size may also represent a reduction in threshold for flap during the study period, although we are cautious in drawing conclusions based on this non-statistically significant result.

This study was limited by its design as a retrospective, observational, single armed study with no comparison group. The patient cohort was operated on by a specific experienced surgical team with a subspecialist interest in sarcoma surgery. Results may vary according to surgical expertise and experience. Potential selection bias and possible shifts in clinical practice during the study period confound our ability to make definitive recommendations regarding the indications for flaps.

## Conclusion

This study demonstrates a low rate of MWCs and reoperation for irradiated sarcoma wounds reconstructed with soft tissue flaps by a single surgical team. Flap reconstruction is resource intensive with potential for flap related morbidity. Further prospective studies are required to confirm the utility of flaps, the generalizability of the approach and to clearly define the indications for free and pedicled soft tissue transfer.

## Ethics Statement

This study was carried out in accordance with the recommendations of the National Statement on the Ethical Conduct of Human Research (NHMRC; 2007). Written and informed consent was not required. The protocol was approved by the Human Research Ethics Committee (HREC)-A, St. Vincent’s Hospital, Melbourne (Ref: QA 106/15).

## Author Contributions

LC contributed to the design of the study, collected and analyzed data and prepared the manuscript. JI assisted in analysis of data and preparation of the manuscript. DG and PC provided clinical cases, contributed to the design of the study, and assisted in preparation of the manuscript. All authors agreed to be accountable for the content of the work.

## Conflict of Interest Statement

The authors declare that the research was conducted in the absence of any commercial or financial relationships that could be construed as a potential conflict of interest. The reviewer TD and handling Editor declared their shared affiliation.
